# Amyloid Triggers Extensive Cerebral Angiogenesis Causing Blood Brain Barrier Permeability and Hypervascularity in Alzheimer's Disease

**DOI:** 10.1371/journal.pone.0023789

**Published:** 2011-08-31

**Authors:** Kaan E. Biron, Dara L. Dickstein, Rayshad Gopaul, Wilfred A. Jefferies

**Affiliations:** 1 Department of Microbiology and Immunology, University of British Columbia, Vancouver, British Columbia, Canada; 2 The Biomedical Research Centre, University of British Columbia, Vancouver, British Columbia, Canada; 3 Michael Smith Laboratories, The University of British Columbia, Vancouver, British Columbia, Canada; 4 Fishberg Department of Neuroscience and Friedman Brain Institute, Mount Sinai School of Medicine, New York, New York, United States of America; 5 Department of Zoology, University of British Columbia, Vancouver, British Columbia, Canada; 6 Department of Medical Genetics, The University of British Columbia, Vancouver, British Columbia, Canada; University of Michigan School of Medicine, United States of America

## Abstract

Evidence of reduced blood-brain barrier (BBB) integrity preceding other Alzheimer's disease (AD) pathology provides a strong link between cerebrovascular angiopathy and AD. However, the “Vascular hypothesis”, holds that BBB leakiness in AD is likely due to hypoxia and neuroinflammation leading to vascular deterioration and apoptosis. We propose an alternative hypothesis: amyloidogenesis promotes extensive neoangiogenesis leading to increased vascular permeability and subsequent hypervascularization in AD. Cerebrovascular integrity was characterized in Tg2576 AD model mice that overexpress the human amyloid precursor protein (APP) containing the double missense mutations, APPsw, found in a Swedish family, that causes early-onset AD. The expression of tight junction (TJ) proteins, occludin and ZO-1, were examined in conjunction with markers of apoptosis and angiogenesis. In aged Tg2576 AD mice, a significant increase in the incidence of disrupted TJs, compared to age matched wild-type littermates and young mice of both genotypes, was directly linked to an increased microvascular density but not apoptosis, which strongly supports amyloidogenic triggered hypervascularity as the basis for BBB disruption. Hypervascularity in human patients was corroborated in a comparison of postmortem brain tissues from AD and controls. Our results demonstrate that amylodogenesis mediates BBB disruption and leakiness through promoting neoangiogenesis and hypervascularity, resulting in the redistribution of TJs that maintain the barrier and thus, provides a new paradigm for integrating vascular remodeling with the pathophysiology observed in AD. Thus the extensive angiogenesis identified in AD brain, exhibits parallels to the neovascularity evident in the pathophysiology of other diseases such as age-related macular degeneration.

## Introduction

Alzheimer's disease (AD) is a neurodegenerative disorder and is the leading cause of dementia in the elderly [1]. AD is characterized by the presence of extracellular neuritic plaques comprised of the amyloid-beta (abeta) peptide and intracellular neurofibrillary tangles comprised of the tau protein [2], [3]. Abeta peptides are produced from the proteolytic cleavage of the amyloid precursor protein (APP). The amyloid cascade hypothesis implicates abeta as the key player in the formation of senile plaques and neuronal death [4]. The strong link between cerebrovascular pathology and AD was historically overlooked since occurrences of stroke prior to the exhibition of AD-like symptoms excluded patients from being diagnosed with AD [5]. However, recent studies have found that vascular risk factors and neurovascular dysfunction associated with hypotension, hypertension [6], [7], cholesterol levels, type II diabetes mellitus [8], smoking [9] and oxidative stress [10] play integral roles in the pathogenesis of AD [11]. In addition, genetic factors including the apolipoprotein E genotype and an associated polymorphism in the ATP-binding cassette A1 lipid transporter indicate a direct link between AD and vascular disease [12]. Furthermore, approximately 90% of AD patients have cerebrovascular amyloid angiopathy (CAA), consisting of abeta in small arteries, arterioles and capillaries [13]. CAA is also present in 50% of the population over the age of 90 years [13]. Vascular accumulation of abeta species 1–42 and abeta species 1–40 in AD is becoming a well-documented risk factor for cerebral hemorrhage, atherosclerosis and arteriosclerosis [14], [15], [16]. CAA involves the deposition of abeta within the small blood vessels of the brain and is found within the walls of the leptomeninges and parenchymal arteries, arterioles and capillaries [17], [18]. Thickening of arteriole walls and formation of microaneurysms occur concurrently in CAA. CAA is associated with degeneration of smooth muscle cells, ischemic white matter damage, fibrinoid necrosis and dementia [19]. Additionally, the E22Q (Dutch), and E22G (Arctic) mutants produce vasculotropic variants peptides of abeta 1–40 that contribute distinct hereditary phenotypes of cerebral amyloid angiopathy, manifesting varying degrees of brain vessels tropism and cytotoxic effects, and impaired microvessel remodeling and angiogenesis [20] that differentially activate mitochondrial apoptotic pathways [21].

Blood brain barrier (BBB) dysfunction was initially identified in animal models of AD [22] and was later confirmed as a prominent, though unexplained, clinical feature of AD in patients [23]. The origin of BBB dysfunction during AD is not known, but APP expression leading to the generation of abeta may be directly involved in this process as BBB leakiness has been demonstrated in a number of AD transgenic animals models in which forms of APP are overexpressed, including the Tg2576 that overexpresses the human APP695 containing the double missense Swedish mutations (K670N/M671L) that causes a form of early-onset AD [22], [24], [25], [26]. We have previously demonstrated that BBB integrity is compromised in this mouse model as early as 4 months of age; long before other disease pathology occur, such as consolidated amyloid plaques [22]. These and other studies that assessed BBB integrity by examining serum protein leakage and dyes extravasation have shown that BBB disruption is subtle, transient, and consistent in older mice [22], [24], [25], [26], however the exact pathophysiology that leads to BBB leakiness is still unclear. In addition, we have also shown that vaccination with abeta can reverse BBB pathology [26]. Furthermore, our animal studies are complimented by magnetic resonance imaging (MRI) and related *in vivo* imaging studies [23] that show BBB permeability is increased in humans affected with AD. Overall, our previous data and those from other studies are consistent with the current “vascular hypothesis” in which vascular damage is caused by reduced blood perfusion of the brain leading to hypoxia, and BBB dysfunction and subsequent neurodegenerative changes such as the accumulation of abeta [27], [28], neuroinflammation [29], and the eventually breakdown of the neurovascular unit [30] resulting in vascular death. Under hypoxia, hypoxia-inducible factors initiate angiogenesis through the upregulation of angiogenic factors, such as vascular endothelial growth factor (VEGF). Angiogenesis, in this model, would be required to ensure tissue regeneration but would likely be limited to replacing the damaged tissues and ensuring oxygenation of brain tissues. The “vascular hypothesis” is becoming an increasingly important area of clinical and scientific exploration; however, it is interesting to consider alternative explanations that are consistent with the existing body of evidence.

The BBB refers to the extensive network of capillary blood vessels, comprised of highly specialized endothelial cells, which compartmentalize the brain from the peripheral blood. The physical seal of the BBB is maintained by several different inter-endothelial tight junctional complexes (reviewed by [31], [32]) comprised of a variety of plasma membrane spanning proteins (like occludin), scaffold cytoplasmic proteins (like ZO-1) and the actin cytoskeleton. Therefore, it is possible that amyloidogenesis influences BBB integrity in AD at the level of the tight junction (TJ) proteins that maintain the barrier. Here we directly address the hypotheses that mechanisms that influence the BBB disruption are due to vascular cell death caused by apoptosis, consistent with the “vascular hypothesis” of AD or endothelial cell proliferation during angiogenesis resulting in hypervascularity in AD.

Our objective was to characterize the relationship between amyloidogenesis and BBB integrity through changes in TJ morphology in the Tg2576 AD mouse and thereby, identify a mechanism that can explain BBB disruption in AD. We report that the Tg2576 AD mice exhibit no apparent vascular apoptosis but have significant TJ disruption, which is directly related to neoangiogenesis leading to a significant increase in vascular density in AD brain. Establishing hypervascularization as a mechanistic explanation for amyloid associated TJ pathology provides new modalities for therapeutic intervention that target the restoration of the BBB by modulating angiogenesis, thereby possibly prevent AD onset and potentially repairing damage in the AD brain.

## Materials and Methods

### Ethics Statement

All animal procedures were conducted with approval by the University of British Columbia Animal Care Committee under the direction of the Canadian Council for Animal Care.

### Mice

Tg2576 transgenic (Tg/+) mice were used in this study. These mice express human APP695 containing the Swedish missense mutations (K670N/M671L) or APPsw [33], under control of the hamster prion protein promoter (Taconic). Mice were maintained on mixed C57Bl6/SJL background by mating heterozygous Tg2576 males to C57Bl6/SJL F1 females. Wild-type (+/+) littermates were used as controls. Aged mice were 18 to 24 months of age while young mice were five months of age. Mice were fed standard lab chow and water *ad libitum* and kept under a 12 hour light/dark cycle.

### Classification of Human Brain Tissues

Well-characterized tissue reference standards of postmortem medial cortical and hippocampal brain tissues were obtained from the Kinsmen Laboratory Brain Bank at the University of British Columbia (Vancouver, BC, Canada). Reference standards of brains from no disease (ND) and from AD patients were used. Classification of clinical and pathological histories of the respective patients were as described in [34].

### Tissue preparation

Mice were terminally anesthetized with Avertin (0.0 2 mL/1 g). Brains were rapidly excised, olfactory bulbs removed and post-fixed in 4% paraformaldehyde for four days at 4°C. The brains were then imbedded in paraffin and sectioned serially at 5 µm. Paraffin embedding, sectioning, and dewaxing were performed by Wax-it Histology Services Inc. (Vancouver, BC, Canada). For immunoblotting, brains were rapidly excised and the hippocampus and neocortex was isolated from both brain hemispheres using methods demonstrated by Hagihara *et al*. [35].

### Immunostaining

Dewaxed paraffin mice brain sections underwent antigen retrieval using a conventional stovetop pressure cooker using 20 mM Tris with 0.7 mM EDTA buffer (pH 9.0) at full steam for 2 minutes. Cooled slides were then incubated in blocking buffer (25% normal goat serum; 3% BSA; 0.3% Triton X-100, Sigma) for 1 hour at room temperature. Primary antibodies used included rabbit anti-ZO-1 (1∶200, Invitrogen), mouse anti-ZO-1 (1∶200, Invitrogen), rabbit anti-occludin (1∶200, Invitrogen), rabbit anti-activated caspase-3 (1∶1000, Imgenex), and mouse anti-human CD105 (1∶20, DAKO). Primary antibody staining was performed overnight at 4°C in staining buffer (10% normal goat serum; 3% BSA; 0.3% Triton X-100). Normal donkey serum was used when staining with goat primary antibodies. Secondary antibodies used were complimentary to the species of the primary conjugated with either Alexa Fluor dyes 488 or 568 (1∶500, Invitrogen). Secondary antibody staining was performed at room temperature for 1 hour in staining buffer. TOTO-3 (1∶10000, Invitrogen) was used for nuclear counterstaining. Sections were washed in PBS with 0.1% Tween-20 (Sigma) three times for 5 minutes each between staining steps. Stained sections were coverslipped using Fluoromount-G (Southern Biotech) and allowed to air dry in the dark overnight.

Human brain tissues were stained using methods described by [34]. Briefly, free-floating 30 µm thick sections were immunolabelled with an anti-laminin primary antibody (1∶100, rabbit, Sigma). Sections were then treated with the appropriate biotinylated secondary antibodies (1∶1000; DAKO) for 1 hour at room temperature, followed by incubation in avidin-biotinylated horseradish peroxidase complex (1∶1000; ABC Elite, Vector Labs). Peroxidase labeling was visualized by incubation in 0.01% 3,3-diaminobenzidine (DAB; Sigma) solution. When a dark purple/black color developed, sections were washed, mounted on glass slides, air-dried and coverslipped with Entellan (EMD Biosciences).

### Confocal and quantitative analysis of tight junction morphology

Brain sections were analyzed from paraffin blocks from every fifth section. Images, taken on a Zeiss LSM510 Meta (Zeiss, Germany), were acquired with 16 slices, averaged four times, through the Z-plane using a 40×/1.3 oil-immersion Plan-Neoflaur objective. The composite projected image was imported into Adobe Photoshop at 600 dpi and optimized for contrast and brightness. Quantitative analysis of tight junction morphology was analyzed according to the methodology developed by Plumb *et al*. [36]. Confocal data sets represented approximately 100 cerebral blood vessels from both young and aged Tg2576 and littermate controls in the frontal cortex and hippocampus. Individual vessels were scored as either normal (1) or abnormal (0) for ZO-1 expression. Normal ZO-1 expression was judged as strong, continuous, intense and linear staining. In contrast, abnormal ZO-1 expression was judged as weak, punctate and/or discontinuous staining. Abnormal ZO-1 blood vessel expression was compared to normal blood vessels found in normal control or in normal vessels in diseased brains. To minimize the recording of incomplete or undulating vessels as abnormal due to observed “gaps” in ZO-1 staining, evidence of vessel continuity was sought in the images. For example, the presence of stained nuclei (with TOTO-3) or punctate or diffuse ZO-1 remnants was used to localize the position of abnormal gaps along the vessel tract. The incidence of tight junction disruption was defined as the average percentage of blood vessels in a given region of brain that displayed abnormal tight junction morphology.

### Microvessel density quantification in Mouse Tissues

Microvessel density (MVD) was quantified by confocal microscopy using the methods developed by Guo *et al.* [37] with minor modifications. Using CD105 as a marker of angiogenic cerebrovasculature [38], images of optimal fluorescent intensity were acquired and analyzed using the Zeiss LSM510 Meta software. Areas within the brain section containing high density (“hotspots”) [39] CD105 staining were imaged using the 20×/0.45 N-Achroplan objective using the confocal imaging parameters mentioned previously. The total fluorescence area (TFA) in µm^2^ was integrated above background, by the software, for each hotspot. The average TFA from four different hotspots per mouse was quantified. The TFA was used as a numerical representation of the total microvessels stained by the CD105 antibody. The MVD of the imaged field was expressed as a ratio of the TFA to the total area of the image.

### Microvessel density quantification in Human Tissues

MVD was quantified using the hotspot method, similar to how the MVD in the mice were quantified. Briefly, areas within a brain section containing high density (“hotspots”) [13] laminin staining were imaged using a Olympus LCPlanFL 20×/0.40 Objective on a Zeiss Axioplan-2 light microscope equipped with a DVC camera (Diagnostic Instruments). MVD quantification was performed using ImageJ (Rasband, W.S., ImageJ v1.44p, U. S. National Institutes of Health, Bethesda, Maryland, USA, http://rsb.info.nih.gov/ij/, 1997–2011). A background threshold level was initially determined from an 8-bit grey scale, derived from an original black and white image, in control slides processed without primary antibody. The percentage of pixels having a staining intensity greater than the corresponding threshold was then used to integrate the percentage area occupied by laminin staining for each hotspot. The average percentage area occupied by laminin staining from four different hotspots per brain region per patient was quantified. The MVD of the imaged field was expressed as a percent area occupied by the laminin staining to the total area of the image.

### Quantitative western blot analysis

Isolated neocortex and hippocampal tissues were homogenized in 1% NP-40 lysis buffer (20 mM Tris-base (pH 8.8), 2 mM EDTA, 150 mM NaCl, 1% NP-40 with protease inhibitors (Roche)) using a QIAGEN TissueLyser II set at 19 Hz for 20 minutes. To shear genomic DNA, homogenized samples were passed ten times through a 21-gauge needle then incubated on ice for 30 minutes. The homogenate was centrifuged at 4°C at 14000×g for 30 minutes. Protein concentrations from the supernatants were determined by BCA assay (Pierce) and samples were adjusted to final concentration of 30 µg per lane. Proteins were resolved in 10% SDS-PAGE gels according to standard practices [40]. Immunoblotting was performed on nitrocellulose membranes (Pall) using rabbit anti-occludin (1∶1000, Invitrogen), mouse anti-human CD105 (1∶1000, DAKO) and mouse anti-beta actin (1∶1000, Santa Cruz). Alexa Fluor 680-conjugated anti-rabbit IgG (Invitrogen) and IRDye800 conjugated anti-mouse IgG (Rockland) were used as secondary antibodies. All antibody dilutions were made in milk protein solutions. Signal intensities were analyzed by using the Odyssey infrared image system (LICOR).

### Statistical analysis

All experiments were performed at least three times in triplicate. Statistical comparisons of data between aged and young Tg2576 AD mice and wild-type control littermates were performed with either Student's t-test or 2-way ANOVA for unmatched values with Bonferroni post-tests. All statistical analyses were performed using GraphPad Prism (v5.01 for Windows, GraphPad Software, San Diego California USA, www.graphpad.com). *p*-values less than 0.05 were considered significant. Values are expressed as mean ± SEM.

## Results

### Tg2576 mice have a higher incidence of abnormal cerebrovascular tight junction morphology

**Figure 1:**
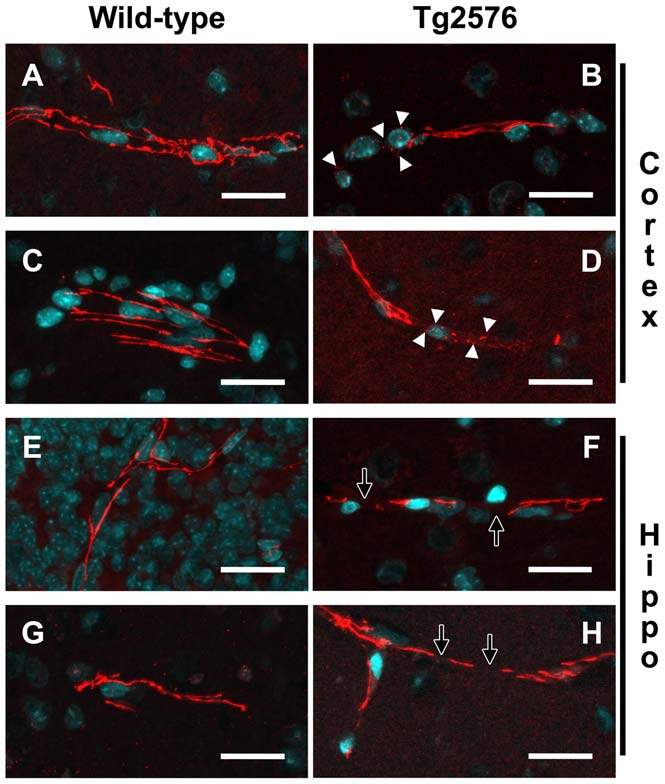
Tg2576 AD mice have cerebral tight junction pathology. Representative confocal micrographs of cerebral blood vessels from aged Tg2576 and wild-type mice immunolabeled for either occludin or ZO-1 (red) and counterstained for DNA (blue) with TOTO-3. Blood vessels, imaged in the neocortex and hippocampus, which exhibited strong, continuous and linear occludin (A and C) or ZO-1 (E and G) expression were considered normal, as demonstrated in the wild-type. Abnormal occludin (B and F) and ZO-1 (D and H) staining displayed punctate (white arrowheads), discontinuous or interrupted (hollow white arrows), as seen in the Tg2576 cerebrovasculature. Results are representative from three mice per group from three separate experiments. Scale bar represents 20 µm.

To assess changes in TJ morphology we examined the staining patterns of occludin and ZO-1, both well-established TJ markers, by confocal microscopy. TJ morphology was characterized in several brain regions including the neocortex, hippocampus and choroid plexus of wild-type and Tg2576 mice. Nearly all the observed blood vessels were sectioned longitudinally. Transverse blood vessels were rare. Strong, continuous and linear staining patterns of occludin and ZO-1 within the cerebrovasculature were considered normal, which was indistinguishable in both the cortex or hippocampus regardless of age or genotype [[**Figure 1: A, C, E and G**]]. The faint outline of the vessel track, enhanced by nuclear counterstaining, allowed junctional abnormalities to be easily spotted. Compared to controls, Tg2576 mice exhibited a higher incidence of punctate staining of occludin [[**Figure 1: B and F**]] and ZO-1 [[**Figure 1: D and H**]] in the neocortex and hippocampus, respectively. The choroid plexus, a region of the brain not affected in AD, exhibited normal TJ patterns for both occludin and ZO-1 in all mice (data not shown).

**Figure 2:**
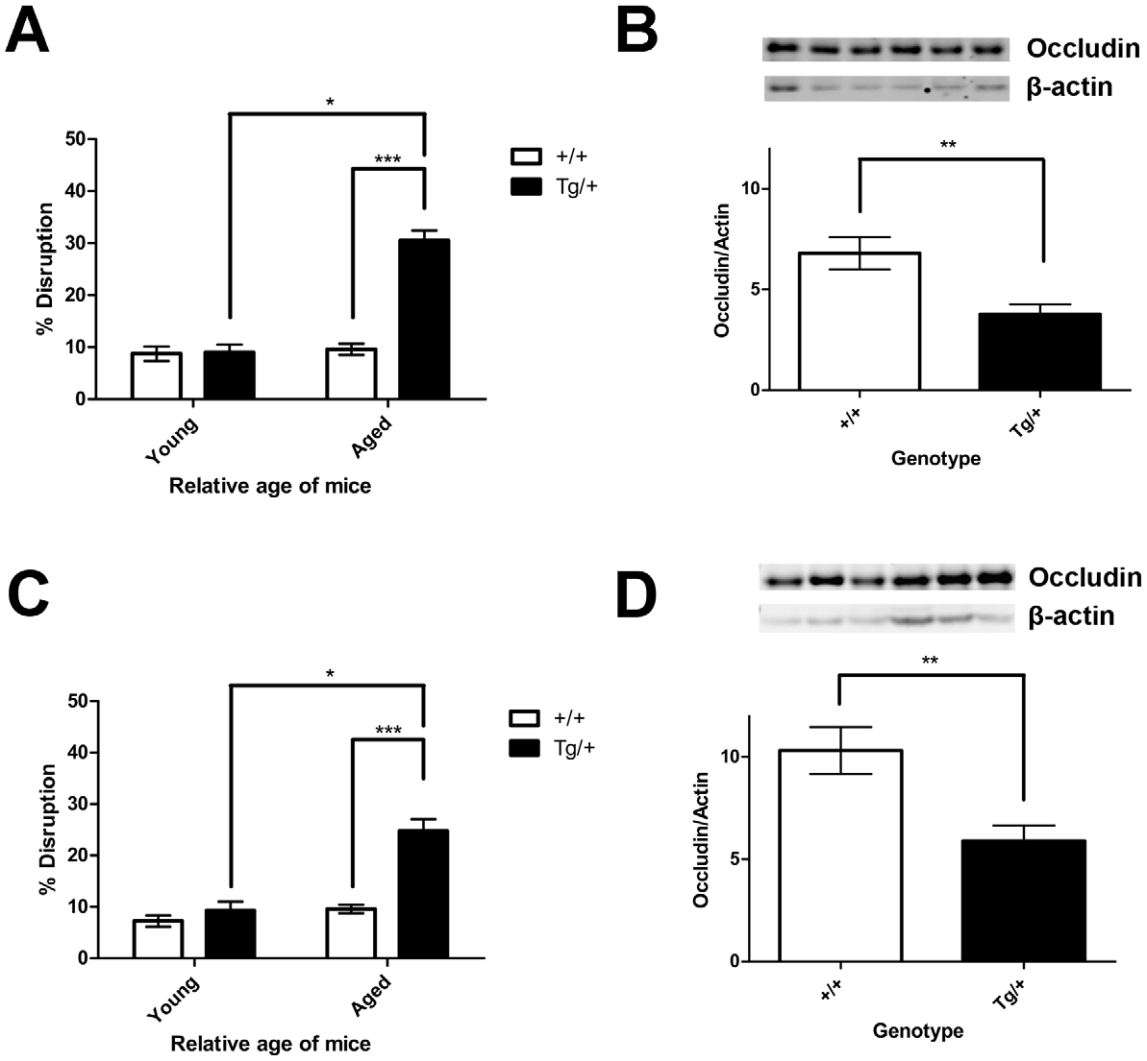
Aged Tg2576 Mice have reduced tight function expression. The expression of occludin or ZO-1 were compared quantitatively between wild-type and Tg2576 mice in the neocortex (A and B) and hippocampus (C and D). (A) The percentage of cortical cerebral blood vessels with abnormal ZO-1 expression patterns was significantly higher in the aged Tg2576 mice compared to age-matched wild-type (***p<0.001). The incidence of ZO-1 disruption was also significantly higher in aged Tg2576 mice compared to young Tg2576 (Young wild-type, n = 4; Young Tg2576, n = 3; aged wild-type, n = 5; aged Tg2576, n = 4; *p<0.05) in the cortex. (B) Aged Tg2576 mice had significantly reduced occludin protein levels in the cortex compared to age-matched wild-type (n = 7, **p = 0.0072). (C) The percentage of hippocampal cerebral blood vessels with abnormal ZO-1 expression patterns was significantly higher in the aged Tg2576 mice compared to age-matched wild-type (***p<0.001). Similarly, the incidence of ZO-1 disruption was also significantly higher in aged Tg2576 mice compared to young Tg2576 (Young wild-type, n = 4; Young Tg2576, n = 3; aged wild-type, n = 5; aged Tg2576, n = 4; *p<0.05) in the hippocampus. (D) Aged Tg2576 mice had significantly reduced occludin protein levels in the hippocampus compared to age-matched wild-type (n = 7, **p = 0.0076). Values represent mean ± SEM.

We then quantified the TJ disruption by calculating the average percentage of blood vessels displaying abnormal morphology. The neocortex of aged Tg2576 mice showed a significant increase in TJ disruption (30.50±1.94%; ***p<0.001, 2-way ANOVA) compared to wild-type littermates (which averaged 10%) [[**Figure 2: A**]]. There was also a significant difference in the incidence of TJ disruption between aged Tg2576 and young, Tg2576 mice (*p<0.05, 2-way ANOVA) [[**Figure 2: A**]].

Similar results were seen in the hippocampus. Aged Tg2576 mice (24.75±2.32%; ***p<0.001, 2-way ANOVA) showed a significant increase in the incidence of TJ disruption compared to wild-type littermates (averaging 10%) [[**Figure 2: C**]]. A significant increase in the incidence of TJ disruption was seen in aged Tg2576 mice compared to young Tg2576 mice (*p<0.05, 2-way ANOVA) [[**Figure 2: C**]]. The amount of TJ disruption in young mice, in both the cortex and hippocampus, of both genotypes averaged approximately 10% and was not significant.

Occludin protein levels were examined by western blot in the neocortex and hippocampus of aged Tg2576 mice. In the cortex, the ratio of occludin to β-actin was reduced by nearly half [[**Figure 2: B**]] in the transgenic mice (3.78±0.49) compared to the wild-type (6.81±0.80, **p = 0.0072, t-test). The hippocampus exhibited a similar reduction in the ratio of occludin to β-actin in the aged Tg2576 mice (5.89±0.76) compared to the wild-type (10.31±1.15, **p = 0.0076, t-test) [[**Figure 2: D**]].

### Aged Tg2576 brains have limited apoptotic and increased angiogenic cerebral vascular signals

**Figure 3:**
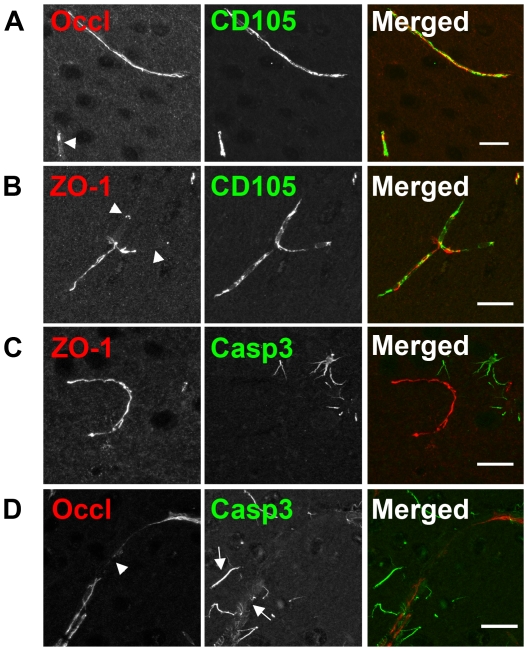
Angiogenesis not apoptosis induces alterations in tight junction immunoreactivity in Tg2576 mice. Representative confocal micrographs of TJs (ZO-1), double stained for markers of angiogenesis or apoptosis in aged wild-type and Tg2576 mice. All vessels stained for CD105 regardless of the TJ expression pattern. White arrowheads point to regions of TJ abnormality in the vasculature. Double staining of blood vessels with ZO-1 (red) and CD105 (green) in wild-type (A). and Tg2576 (B) neocortex. Double staining of vessels with ZO-1 (red) and caspase 3 (green) in wild-type (C) and Tg2576 (D) neocortex. Caspase-3 staining did not colocalize with ZO-1 staining indicating an absence of apoptosis in the vasculature. Results are representative of three separate experiments of three mice per group of brain tissues examined. Scale bar represents 20 µm.

Apoptosis and angiogenesis were explored as possible mechanisms to explain the observed brain vascular TJ abnormalities in the aged Tg2576 mice. We used activated caspase-3 as a marker of apoptosis [[**Figure 3: C–D**]]. In all examined sections, there was no activated caspase-3 immunoreactivity in endothelial cells. Activated caspase-3 staining was noted in other cell types and had a filamentous-like morphology. In young and aged wild-type mice, there was very limited activated caspase-3 staining within the neocortex [[**Figure 3: C**]]. Blood vessels in the cortex and hippocampus that did exhibit TJ abnormalities, [[**Figure 3: D**]], had a filamentous-like staining morphology that was directly adjacent to or overlapped with blood vessels [[**Figure 3: D** (**white arrows**)]]. All mice had hippocampal staining of activated caspase-3 in the CA1, CA2, CA3 and DG regions to various degrees. Young Tg2576 and wild-type (both young and aged) had significantly higher activated caspase-3 staining densities within the hippocampus compared the cortex. The overall density of caspase-3 hippocampal staining in young Tg2576 and wild-type (all ages) mice was noted to be lower than aged Tg2576 mice. However, aged Tg2576 mice had significant activated caspase-3 staining within the both the neocortex and hippocampus that tended to center around probable plaque locations.

To assess for angiogenesis we used CD105, a well establish endothelial marker [41]. CD105 stained all vessels uniformly, regardless of age and genotype. However, a higher proportion of blood vessels were stained with CD105 in aged Tg2576 compared to age-matched wild-type littermates and young mice of both genotypes [[**Figure 3: B**]].

### Aged Tg2576 mice have increased microvascular density

**Figure 4:**
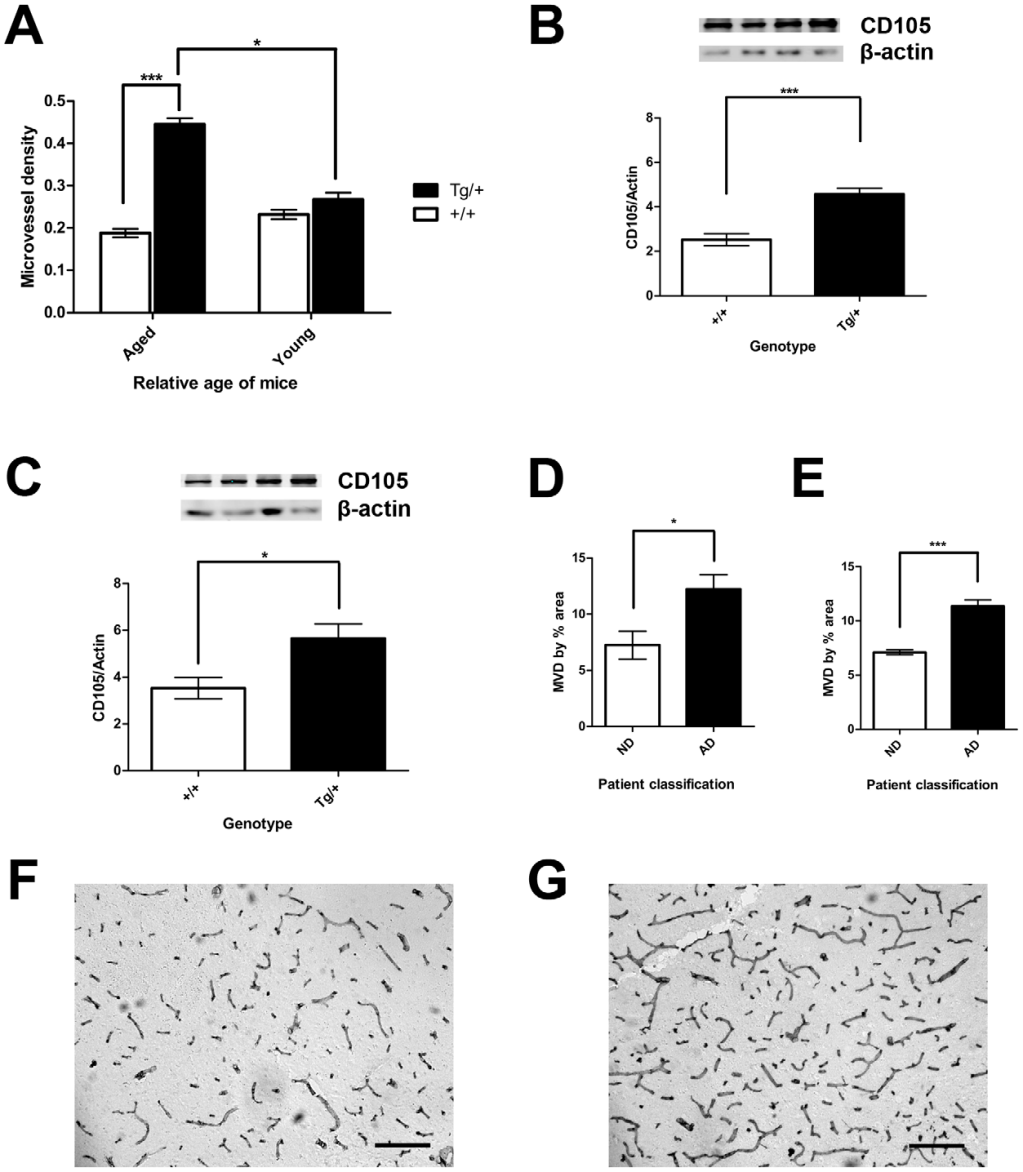
Microvascular Density is increased in Aged Tg2576 and in Human patients with AD. The MVD, by CD105 staining, in the cerebrovasculature and CD105 protein expression were quantified in aged and young Tg2576 and wild-type. (A) Aged Tg2576 mice had a significantly higher MVD compared to age-matched wild-type (***p<0.001). Aged Tg2576 were had a significantly higher MVD compared to young Tg2576 (Aged wild-type, n = 5; aged Tg2576, n = 4; young wild-type, n = 4; young Tg2576, n = 3; *p<0.05). Although not significant, young Tg2576 mice trended to a higher average MVD compared to wild-type. (B) Aged Tg2576 mice had a significantly increased CD105 protein levels in the cortex compared to age-matched wild-type (wild-type, n = 5; Tg2576, n = 6; ***p<0.001). (C) Aged Tg2576 mice had a significantly increased CD105 protein levels in the hippocampus compared to age-matched wild-type (n = 7, *p<0.05). (D) The cortex of the AD patient had a significantly increased MVD, as measured by % area occupied by laminin staining, compared to the ND patient (n = 4, *p<0.05). (E) The hippocampus of the AD patient had a significantly increased MVD, as measured by % area occupied by laminin staining, compared to the ND patient (n = 4, ***p<0.001). Representative images of immunohistochemical staining for laminin in the cortex of the ND patient (F) and the AD patient (G). Scale bar represents 95 µm. Values represent mean ± SEM.

It was noted earlier that a higher proportion of blood vessels were stained with CD105 in aged Tg2576mice. The microvascular density (MVD) was quantified in the brains of young and aged Tg2576 mice and age-matched wild-type littermates by CD105 staining. The MVD was defined as a ratio of the TFA to the total area of an imaged field and was used as a surrogate measure of angiogenesis. Aged Tg2576 mice had a significantly higher MVD compared to wild-type. The average MVD in aged Tg2576 mice (0.4453±0.0146; ***p<0.001, 2-way ANOVA) was over double that of wild-type (0.1882±0.0010) [[**Figure 4: A**]]. When compared to young Tg2576 mice, the MVD in aged Tg2576 was over 1.5 times (*p<0.05, 2-way ANOVA). Young Tg2576 mice had a trend towards a higher average MVD (0.2674±0.0161) but the differences were not significantly different compared to wild-type (0.2321±0.0110) [[**Figure 4: A**]].

CD105 protein expression levels were quantified by western blot in both the neocortex and hippocampus of aged Tg2576 and wild-type mice. In the cortex, the ratio of CD105 to β-actin was nearly double [[**Figure 4: B**]] in the Tg2576 mice (4.57±0.27) compared to that of the wild-type (2.52±0.27, ***p<0.001, t-test). The hippocampus exhibited a similar increase in the ratio of CD105 to β-actin in the aged Tg2576 mice (5.66±0.62) compared to the wild-type (3.53±0.46, *p<0.05, t-test) [[**Figure 4: C**]].

In a preliminary examination of human tissues, quantifying the amount of laminin staining in the brains of no disease (ND) and AD patients was conducted in carefully validated cases from the Kinsmen Laboratory Brain Bank at University of British Columbia. These samples were used as reference standards of postmortem medial cortical and hippocampal brain tissues [34] to provide support that increased cerebral vascular density also extends from animal models of AD into clinical disease in humans. In the cortex of the AD reference standard, the average MVD by percent area was nearly double (12.23±1.28%, *p<0.05, t-test) compared to the ND patient (7.25±1.25%) [[**Figure 4: D**]]. Similarly, the hippocampus of the AD reference displayed a doubling in the average MVD (11.35±0.60%, ***p<0.001, t-test) compared to the ND reference (7.10±0.23%) [[**Figure 4: E**]]. The extent of the increased cortical cerebral vascular density from laminin staining in the AD patient [[**Figure 4: G**]] can be visually seen in the representative images compared to the ND patient control [[**Figure 4: F**]. Hippocampal tissue displayed similar staining patterns in the AD and ND patients (data not shown). Additional work is needed to further extend these observations in populations of AD patients and controls.

## Discussion

Reduced BBB integrity precedes other AD neuropathology such as amyloid plaques. However, the current dogma holds that BBB leakiness in AD is likely due to vascular deterioration and apoptosis resulting from brain hypoxia. The goal of this study was to test the “Vascular hypothesis” of AD [30] that would require evidence of cerebrovascular damage and apoptosis, or the alternative hypothesis, that amyloidogenesis leads directly to angiogenesis and hypervascularization that underlie increased vascular permeability in AD. We have previously established by quantification using the Evans Blue dye method, that the BBB is compromised in the Tg2576 AD model mouse that express a human APP695 transgene containing the Swedish missense mutations (K670N/M671L) or APPsw [33], under control of the hamster prion protein promoter. We also demonstrated that the breakdown of the BBB can be rescued by immunization with abeta [22], [26]. In the present study, the integrity of the BBB was assessed by examining TJ morphology in Tg2576 AD mice that express the human APP containing the double missense mutations, found in a Swedish family, that causes early-onset AD. Two separate age groups of mice were examined: five months old (prior to disease onset) and aged 18+ months old (well-after disease onset). Aged Tg2576 mice were found to have significantly abnormal TJ expression, compared to controls, which correlated with increased angiogenesis and hypervascularization. Taken together, the expression of APPsw influences angiogenesis that appears to cause BBB disruption in Tg2576 mice at the level of TJs. This study is believed to be the first comprehensive examination of TJ related BBB dysfunction in AD and is important in the characterization the pathophysiology of AD brain.

Our data do not support the existing “vascular hypothesis” of AD and rather support the concept that amyloidogenesis leads directly to angiogenesis rather than through a model requiring hypoxia, vascular damage and subsequent vascular repair. During amyloidogenesis, abeta peptides arise by successive cleavage of unprocessed APP by beta-and gamma-secretases [42], [43], [44], [45], [46], [47] and recently, it has come into question whether the large aggregated extracellular abeta plaques, a hallmark of AD pathology, directly cause the neurodegenerative effects in AD. It is emerging that the smaller, more toxic, soluble abeta oligomers may directly initiate disease (reviewed by [48]). It has been suggested that the presence of these toxic oligomers could negatively influence endothelial survival and TJ expression. However, our data is consistent with the possiblity that vasculotropic peptides of abeta generated during amyloidogenesis contribute to cerebral amyloid angiopathy and effect microvessel remodeling and angiogenesis [20], [21]. Several lines of *in vitro* evidence have explored endothelial dysfunction by abeta. Marco *et al.* [49] demonstrated that abeta 1–42 stimulated endothelial cultures induced aberrant expression of TJ proteins including claudins, occludins and ZO-1. Gonzalez *et al.* [50] demonstrated that smaller abeta 1–40 aggregates induced endothelial cell permeability and the relocalization of ZO-1 to the cytoplasm. In these studies, abeta induced reactive oxygen species (ROS) was ruled out as a potential cause of BBB leakiness because the presence of ROS detoxifying enzymes did not influence abeta induced damage [51]. However, the reorganization of cytoskeletal proteins was believed to directly influence BBB integrity [51]. Another potential contributor to vascular remodelling in AD is APP itself that has been reported to possess weak ferroxidase activity [52]. Impairment of APP ferroxidase activity in AD could result in ferrous iron overload and result in the formation of superoxide anions and hydroxyl radicals that could result in damage of brain tissues. ROS from other sources including microglial activation, inflammation, from serum leakage, could also effect BBB integrity [53]. However, if this were the case, we would likely be evidence of significant vascular apoptosis in the brains of Tg2576 mice and we can presently find no indication of this.

Another potential mediator of cerebrovascular disruption in AD is a highly toxic dodecameric 56 kDa abeta oligomer, referred to as “abeta*56” that has been directly implicated in memory loss in Tg2576 mice [54]. This oligomer emerges at about six months of age in the Tg2576 mice, when memory deficits first become apparent, but is absent in younger mice [54]. In the present study, five-month old mice had a trend towards increased abnormal vascular TJs. However, Tg2576 mice begin showing a loss of BBB integrity as early as four months of age [22]. During this time, a build-up of smaller less toxic oligomers could begin negatively influencing BBB integrity in this mouse. By six months of age, it is hypothesized that the toxic presence of abeta*56 initiates a cascade of the pathological events associated with the Tg2576 mouse. Although, between the ages of six to 13 months, the relative levels of abeta*56 remain constant in the Tg2576 mice [54]. During this time the eventual accumulation of plaques and dystrophic neurons is enough to cause further pathological damage in this mouse. This could explain the dramatic presence of abnormal cerebral vascular TJ expression in the aged Tg2576 mice, but again however, this mechanism would likely be accompanied obvious vascular cell death and we observe not only wide-spread angiogenesis but hypervascuarity in the absence of endothelial apoptosis. In the context of the present study, it will be interesting to determine if abeta*56 exhibits pro-angiogenic activity suggested by our studies rather than displaying toxic effects towards cerebrovasculature.

In contrast to the studies summarized above that contemplate amyloidogenesis resulting in endothelial damage, our study using an antibody against activated caspase-3 failed to detect endothelial apoptotic events. However, young (5 months of age) and aged (18–24 months of age) Tg2576 and corresponding wild-type mice exhibited active caspase-3 expression with non-endothelial staining predominately within the hippocampus. These data are consistent with the absence of wide-spread endothelial cell death. Although, apoptotic endothelial cells were not observed in this study, *in vitro* evidence does suggest that abeta, especially mutations pertaining to the Dutch mutant, have been shown to induce apoptosis in cultured endothelial cells [21], [55], [56]. Clearly, *in vivo* evidence from clinical studies of AD and in other animal models of AD, is needed in order definitely clarify if abeta induced endothelial apoptosis takes place that we cannot detect or whether the absence of endothelial apoptosis observed with the methods used in this study are validated by other approaches.

Angiogenesis was also examined as another potential mechanism for explaining TJ disruption in cerebrovascular endothelium. There are several lines of evidence that suggest angiogenesis occurs during AD. First, neuroinflammation is a pathological feature of AD [57] and is associated with the increase in cytokines, like IL-1β, that are capable of inducing angiogenesis [58]. The pro-angiogenic growth factor VEGF is also induced by these cytokines [59] and is elevated in AD patients [60]. VEGF directly stimulates endothelial proliferation [61]. Abeta peptides themselves have also been shown to have angiogenic properties [62]. Here, CD105 was used to examine angiogenesis in cerebral blood vessels labeled with the TJ markers. The CD105 expression was not able to distinguish vessels with TJ abnormalities from those without [41], however, the apparent pan-endothelial staining of CD105 was extremely useful as it allowed the quantitation of the density of CD105 staining and thus the microvascular density (MVD), in the entire brain. The MVD was used as a surrogate marker for the amount of angiogenesis present within a given area of tissue section. The greater the vascular density, the more angiogenesis is believed to have occurred. Measuring the MVD is not without limitations, which include a lack of standardization and reliance on the operator to minimize bias leading to the loss of objectiveness [63]. Nonetheless, aged Tg2576 mice had nearly double the MVD compared to age-matched wild-type mice. Young Tg2576 mice exhibited no significant differences in MVD but had a trend towards an increase in MVD compared to the controls.

Angiogenesis in the Tg2576 mouse is controversial. Paris *et al* [64] noted the Tg2576 mouse to have limited angiogenesis. The pan endothelial marker PECAM was used to examine angiogenesis in mice up to 17 months of age. Although there is no consensus in the literature as to which is the “best” marker for studying angiogenesis in the brain, PECAM is not favoured as an angiogenic marker as compared to CD105 [65] since PECAM is not limited to pan-endothelial expression as plasma and inflammatory cells are typically immunolabeled also [66]. CD105 on the other hand has been shown to consistently react with endothelials cells [65], especially those undergoing angiogenesis, and does not react with stromal or inflammatory cells [67], [68]. The data presented herein, demonstrates that an increased CD105-related MVD does indicate that angiogenesis and TJ abnormalities are related in the Tg2576 mouse. Pro-angiogenic signals have been detected in the Tg2576 mouse. Elevated VEGF in the cortical tissue of 20 month old Tg2576 mice [69] supports the hypothesis that angiogenesis occurs in this AD model. Finally, increased expression of activation markers associated with angiogenesis of cerebral vasculature has been observed in human AD brains: laminin or von Willerbrand factor expression [34]; integrin αV-β3 expression [70]; and in the APP23 AD mouse model beta3-integrin subunit expression [59]. The conclusions from these studies teach that angiogenesis is exclusively a result of vascular remodeling that takes place as a result of reduced blood perfusion of the brain resulting in hypoxia. Furthermore, BBB dysfunction leads to neurodegenerative changes including accumulation of Aβ [27], [28], neuroinflammation [29] and ensuing breakdown of the neurovascular unit [30] leading to vascular damage and death. We cannot find evidence of cerebral vascular death in the Tg2576 mouse and in contrast, we provide evidence of a extensive neovascularization in the Tg2576 mouse. Furthermore, in a preliminary examination of well-characterized normal and AD reference standards of postmortem medial cortical and hippocampal brain tissues also exhibit significant hypervascularity in AD, far beyond that present in a normal human brain [[**Figure 4: F–G**]].

In many ways, the hypervascularity we report in AD appears to parallel Choroidal neovascularization (CNV) also referred to as the wet form of age-related macular degeneration [71]. It may be wise, therefore, to consider the wider hypothesis that hypervascularization is a cause, not a symptom, of senile dementias, including AD, and perhaps contributes to the pathophysiology of other neurological diseases as well. Therapeutic strategies for AD are desperately needed. It remains to be demonstrated whether modulating AD-related neovascularization will provide a novel treatment for AD. However, given the similarities between the wet form of AMG and AD, future studies can examine this possibility and whether these diseases share biomarkers that can be utilized as aids to diagnose disease, to discover new drugs and to monitor the effectiveness of therapies. Furthermore, vascular related defects have been noted in one of the more advanced experimental therapies for AD that involves mounting an immune response to abeta. Modulating the effects of amyloidogenic-related angiogenesis in relation to anti-abeta immunotherapy could provide novel insights into enhancing current therapeutic approaches.

In summary, this is the first study that indicates that amyloidogenesis leading to AD, regulates and promotes neoangiogenesis that results in extensive disruption of TJ's during vascular growth and proliferation of the BBB. This phenomenon is increasingly manifested with age and with disease severity and results in substantial hypervascularization that is clearly evident in both murine and human forms of AD. Thus, these data support the model that TJ disruption results from increased vascular permeability that takes place during extreme neovascularization in AD, likely triggered by production of APP, abeta or perhaps abeta*56 during amyloidogenesis. These pathophysiological features are profound and severe, appear early in disease development, and may rival neurofibrillary tangles, dystrophic neurons and amyloid plaques as characteristic hallmarks of AD.
